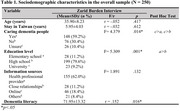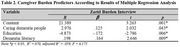# Dementia Literacy on Foreign Caregivers and the Impact of Care Burden

**DOI:** 10.1002/alz70858_097972

**Published:** 2025-12-24

**Authors:** Widiyaningsih, Huei Ling Chiu

**Affiliations:** ^1^ Taipei Medical University, Taipei, Taipei, Taiwan

## Abstract

**Background:**

Dementia, characterized by progressive cognitive and functional decline, poses global challenges and places substantial demands on caregivers. The rising global prevalence of dementia has increased the demand for caregivers in many countries. In Taiwan, dementia prevalence among older adults was 7.99% in 2020. To meet the growing need for dementia care, Taiwan relies heavily on foreign caregivers. By early 2024, 217,000 foreign domestic caregivers were employed, with 76% originating from Indonesia. These caregivers provide essential support for older adults including those with dementia. However, foreign caregivers face significant challenges such as cultural and language barriers and inadequate dementia‐specific training, which exacerbate the caregiver burden among foreign caregivers.

**Purpose:**

To assess dementia literacy and caregiver burden among foreign caregivers and identify the factors influencing caregiver burden.

**Methods:**

A cross‐sectional study was conducted from June to December 2024, involving face‐to‐face interviews with 250 Indonesian caregivers in Taiwan. Convenience sampling was used for participant recruitment. Data were collected using a sociodemographic questionnaire, the Consumer Access, Appraisal, and Application of Services and Information for Dementia (CAAASI‐Dem) to measure dementia literacy, and the Zarid Burden Interview (ZBI) to assess caregiver burden. Statistical analyses included independent sample t‐test and Pearson's Chi‐squared test to evaluate differences between two groups, one‐way ANOVA to compare multiple groups, and multi‐linear regression to identify predictors of caregiver burden.

**Results:**

The mean scores for dementia literacy and caregiver burden were 72.29±13.82 and 30.48±16.44, respectively. Caring for people with dementia, education level, and dementia literacy were significantly associated with caregiver burden (*p* < .05) and identified as significant predictors (*p* < .05).

**Conclusions/Implication for Practice:**

Foreign caregivers exhibited moderate dementia literacy and low caregiver burden. Caring for people with dementia, education level, and dementia literacy emerged as significant predictors of caregiver burden. These findings highlight the need for targeted dementia education programs, particularly for foreign caregivers with lower education levels and those caring for people with dementia, to improve dementia literacy and reduce caregiver burden.